# Transcranial direct current stimulation promotes angiogenesis and improves neurological function via the OXA-TF-AKT/ERK signaling pathway in traumatic brain injury

**DOI:** 10.18632/aging.205724

**Published:** 2024-04-05

**Authors:** Bingkai Ren, Junwei Kang, Yan Wang, Xiangqiang Meng, Ying Huang, Yang Bai, Zhen Feng

**Affiliations:** 1 The Affiliated Rehabilitation Hospital, Jiangxi Medical College, Nanchang University, Nanchang 330003, Jiangxi, China

**Keywords:** tDCS, TBI, angiogenesis, Orexin-A, TF-AKT/ERK signaling pathway

## Abstract

Traumatic brain injury (TBI) and its resulting complications pose a major challenge to global public health, resulting in increased rates of disability and mortality. Cerebrovascular dysfunction is nearly universal in TBI cases and is closely associated with secondary injury after TBI. Transcranial direct current stimulation (tDCS) shows great potential in the treatment of TBI; however, the exact mechanism remains elusive. In this study, we performed *in vivo* and *in vitro* experiments to explore the effects and mechanisms of tDCS in a controlled cortical impact (CCI) rat model simulating TBI. *In vivo* experiments show that tDCS can effectively reduce brain tissue damage, cerebral edema and neurological deficits. The potential mechanism may be that tDCS improves the neurological function of rats by increasing orexin A (OXA) secretion, upregulating the TF-AKT/ERK signaling pathway, and promoting angiogenesis at the injury site. Cellular experiments showed that OXA promoted HUVEC migration and angiogenesis, and these effects were counteracted by the ERK1/2 inhibitor LY3214996. The results of Matrigel experiment *in vivo* showed that TNF-a significantly reduced the ability of HUVEC to form blood vessels, but OXA could rescue the effect of TNF-a on the ability of HUVEC to form blood vessels. However, LY3214996 could inhibit the therapeutic effect of OXA. In summary, our preliminary study demonstrates that tDCS can induce angiogenesis through the OXA-TF-AKT/ERK signaling pathway, thereby improving neurological function in rats with TBI.

## INTRODUCTION

TBI stands as the leading cause of neurological dysfunction and mortality resulting from trauma [1]. The global incidence of TBI surpasses 50 million cases annually [2, 3]. TBI can be categorized into primary and secondary injuries. Primary injury refers to irreversible neurological damage caused by an immediate external force, including brain stem injury, diffuse axonal injury, and cerebral contusion and laceration. On the other hand, secondary injury refers to a progressive nerve injury that ensues due to a cascade of pathophysiological changes following the initial trauma, such as brain edema, cerebral ischemia, and neuronal apoptosis. These secondary injuries can be mitigated or reversed through subsequent therapeutic interventions; therefore, secondary injury is the focus of clinical intervention [4]. The main pathological processes following TBI include cerebral ischemia, vascular dysfunction, calcium iron overload, accumulation of oxygen free radicals, neurotoxic effects of excitatory amino acids, and stimulation of neuroinflammatory factors [5]. Although the mechanisms of action for certain drugs in the treatment of TBI have been preliminarily studied, there are currently no particularly effective drugs that have entered phase III clinical trials. Therefore, research on new therapies capable of reversing neurotrauma after TBI holds significant importance.

The vascular network in the human brain serves as the foundation for cerebral tissue metabolism and function. Cerebrovascular dysfunction is commonly observed in injuries associated with TBI and plays a substantial role in the development of secondary damage following TBI [6]. During the acute phase of TBI, the disruption of cerebral vascular integrity results in increased permeability of the blood-brain barrier. This, in turn, results in vasogenic cerebral edema, intensified brain tissue swelling, elevated intracranial pressure, and subsequently reduced perfusion pressure [7]. During the acute and subacute phases of TBI, the formation of neovascularization in the penumbra region can enhance oxygen and nutrient supply to the injured tissue, thereby promoting TBI recovery and improving brain function [8–10]. Consequently, the early establishment of robust collateral circulation is crucial for the recovery of brain injuries, and promoting angiogenesis may represent a potential therapeutic target for TBI [11]. Orexin-A has been identified as a novel angiogenic peptide, promoting blood vessel formation by activating the MEK/ERK1/2 signaling pathway through Orexin-A receptor activation [12]. Our previous studies have shown that Orexin-A has the potential to ameliorate brain tissue damage in rats with TBI [13]. However, the relationship between Orexin-A and angiogenesis in TBI is still insufficient.

Recently, Transcranial Direct Current Stimulation (tDCS) has garnered significant attention in the field of rehabilitation therapy. tDCS is a non-invasive neurostimulation technique that modulates brain activity by delivering low-intensity direct current through electrodes placed on the scalp. Anodal stimulation enhances excitability in targeted brain regions, thereby promoting neuroplasticity. Conversely, cathodal (−) stimulation suppresses excitability in local brain areas, leading to a reduction in neuronal activity. It has been previously used in the treatment of various brain functional disorders, including depression, Parkinson’s disease, epilepsy, stroke, and TBI. Studies have shown that tDCS can enhance the level of consciousness in patients with post-TBI cognitive impairments [14]. Anodal tDCS, in particular, has been found to improve the cerebrovascular reactivity and dilation of brain parenchymal arterioles, resulting in increased blood flow in the arterioles, improved capillary velocity, augmented functional capillary density, and enhanced tissue oxygenation [15]. It also enhances neuroplasticity in the brain [16]. Preliminary research conducted by our research group has demonstrated significant effects of tDCS on patients with TBI [17], as well as its arousal-promoting effects in comatose rats following TBI. However, the precise therapeutic mechanisms remain not completely comprehended. This paper aims to investigate whether tDCS alleviates neurological damage after TBI and promotes brain injury recovery by promoting brain angiogenesis. Furthermore, it aims to elucidate the role of the OX-A/TF/AKT/ERK signaling pathway in these effects.

## METHODS

### Animals

All animal experiments conducted in this study were subjected to a rigorous review and received approval from the Ethics Committee of The First Affiliated Hospital of Nanchang University (Approval No. CDYFY-IACUC-202302QR058). Moreover, all animal procedures strictly conformed to the guidelines specified in the National Institutes of Health’s Guide for the Care and Use of Laboratory Animals. To ensure consistency, Specific Pathogen Free (SPF) adult male Sprague-Dawley (SD) rats, aged 6 weeks and weighing between 250–300 g, were procured from Zhejiang Weitong Lihua Experimental Animal Technology Co., Ltd. (License No. SCXK (Zhejiang) 2019-0001). All experiments were designed and reported according to the Animal Research: Reporting of *In Vivo* Experiments (ARRIVE) guidelines [18], and every effort has been made to minimize animal suffering. In the first stage of the experiment, a total of 24 rats that met the inclusion and exclusion criteria were randomly divided into 4 groups: sham operation group (*n* = 6), CCI group (*n* = 6), CCI + tDCS group (*n* = 6), CCI + sham-tDCS group (*n* = 6). In the second stage of the experiment, a total of 60 rats that met the inclusion and exclusion criteria were randomly divided into 5 groups: sham operation group (*n* = 12), CCI group (*n* = 12), CCI + sham-tDCS group (*n* = 12), CCI + tDCS group (*n* = 12), CCI + tDCS group+SB334867 (*n* = 12). In the final *in vivo* rat Matrigel plug experiment, 16 SD rats were randomly divided into 4 groups: sham group, TBI group, TBI+OX-A group, and TBI+OX-A+LY3214996 group. The animals were kept in SPF animal laboratory conditions throughout the experiment, maintaining a room temperature of 25°C, humidity is 50% and a 12-hour light-dark cycle. Furthermore, they were given unrestricted access to food and water. Prior to the commencement of the experiment, a 7-day acclimation period was provided to ensure their adaptation to the laboratory environment. The experimental results were obtained by an uninformed researcher.

### TBI rat model

The establishment of the CCI model was described in previous studies [19]. Anesthesia was induced by inhalation of 4% isoflurane (RWD Life Sciences Co., Ltd., Shenzhen, China) and maintained by oral application of 2.5% isoflurane through a mask. Use a heating pad to maintain it at 36.5–37.5°C. Experimental animals were placed in a rat stereotaxic apparatus (RWD Life Sciences Co., Ltd., Shenzhen, China). After the surgical area is prepared and disinfected, the scalp is incised, the subcutaneous tissue and periosteum are peeled off, and a 5.0 mm circular craniectomy is performed using a drill in the left parietal cortex (between bregma and lambda, 1 mm lateral to the midline) without any damage. After craniectomy, a CCI device (YHCI99, Wuhan Yihong Technology Co., Ltd., Wuhan, Hubei Province, China) with a 4.0 mm flat impactor tip was placed in the center of the craniectomy site. Moderate damage was induced at a speed of 4 m/s, a dwell time of 150 ms, and an impact depth of 2.8 mm. The dura mater remained intact over the cortex, rats were excluded if dural integrity was disrupted. Our success rate during modeling in this experiment was about 85%, so we prepared 8 rats per group and 2 spare rats. The sham group received the same treatment except for impaction. The animal was placed on a heated warming pad until the rat regained consciousness and then returned to the cage. In this study, the CCI modeling time was about 15 minutes per animal, and the total anesthesia time was about 20 minutes per animal.

### Transcranial direct current stimulation

TBI rats were treated using an animal-type tDCS device (MBM-IV, Nanchang, Jiangxi, China). The treatment duration was 20 minutes per session, once daily, for a continuous period of 2 weeks, The intensity and duration of each stimulation were selected based on previous research reports [20–23] (i.e., a constant current of 0.5~2.0 mA, each lasting 20–30 minutes is the effective and safe dose range). The specific procedures and treatment parameters were as follows: The tDCS anode electrode was placed around the fronto-parietal region of the rat’s left side, near the site of injury, while the cathode electrode was placed above the right eye socket. In the true stimulation group, the stimulation parameters were as follows: The current intensity gradually increased to 1000 μA within 15 seconds, maintained at a constant direct current of 1 mA for 20 minutes, and then gradually decreased to 0 mA within the final 15 seconds. In the sham stimulation group, the stimulation parameters were as follows: The current intensity gradually increased to 1 mA within 15 seconds, then gradually decreased to 20 μA within the next 15 seconds, maintained at a constant direct current of 20 μA for 20 minutes, and finally, the current intensity gradually decreased to 0 μA within the last 15 seconds. During tDCS treatment, anesthesia was induced by inhalation of 4% isoflurane (Rewid Life Sciences Co., Ltd., Shenzhen, China) and maintained by inhalation of 2.5% isoflurane through a face mask.

### Intraperitoneal drug injection

In our *in vivo* study, we dissolved bromodeoxyuridine (BrdU, Solarbio, China, CAS: 59-14-3, Catalog: B8010) in physiological saline to ensure a final concentration of 10 mg/ml. Starting from the day of CCI surgery until postoperative day 14, we administered Brdu via intraperitoneal injection at a dosage of 50 mg/kg per injection, twice daily. Simultaneously, we dissolved the selective orexin receptor 1 (OXR1) inhibitor SB334867 (ab120164, Abcam, USA) in a 5% DMSO solution, achieving a final concentration of 10 mg/ml. For the tDCS+SB334867 group, we administered SB334867 via intraperitoneal injection at a dose of 30 mg/kg once a day from after CCI surgery and before tDCS treatment until postoperative day 14. The remaining groups received injections of an equivalent volume of 5% DMSO.

### Modified Neurological Severity Scale (mNSS)

The modified Neurological Severity Score (mNSS) is a comprehensive assessment of motor, sensory, balance, and reflex testing. It is conducted following the description provided in previous studies [24, 25]. The scoring ranges from 0 to 18, with higher scores indicating more severe neurological impairment. Each abnormal behavior or untested reflex accounts for 1 point. A total score of 0 indicates normalcy, while scores of 1–6 are classified as mild, 7–12 as moderate, and 13–18 as severe. The mNSS testing is performed before and after TBI at 14 days. All neurobehavioral tests are conducted by two researchers who are unaware of the group assignments.

### Brain water content

It is conducted following the description provided in previous studies [24, 25]. After anesthesia of the rats, the brain tissue was rapidly extracted (*n* = 6 per group) following cardiac perfusion to induce euthanasia. Subsequently, the cerebral tissue was split into left and right hemispheres along the midline using a surgical blade. The wet weight was then obtained by weighing the samples using an electronic balance. Subsequently, the samples were dried in a 100°C oven for 24 hours, followed by reweighing to determine the dry weight. Finally, the brain water content was calculated using the formula: brain water content (%) = (wet weight - dry weight)/wet weight × 100%.

### Blood-brain barrier permeability

To assess blood-brain barrier permeability through the examination of Evans Blue (EB) tracer extravasation into brain tissue, the following procedure was utilized: On the third day after inducing TBI in rats, a 2% EB solution (4 ml/kg) was administered intravenously via the rat's tail vein. After a lapse of four hours, any remaining dye within the vascular system was meticulously removed by perfusing with phosphate-buffered saline (PBS) via the left ventricle. Subsequently, the rats were anesthetized and humanely euthanized. The left hemisphere was promptly weighed and then homogenized in dimethyl sulfoxide (1 ml/100 mg of brain tissue). The homogenized samples were subjected to a 60°C incubation for a duration of 24 hours. Following incubation, they underwent centrifugation at 1000 g for 5 minutes, and the resulting supernatant was collected. Use a multifunctional microplate reader (Varioskan LUX, Thermo Fisher Scientific, USA) to measure the absorbance (OD value) at a wavelength of 620 nm, and a standard curve was concurrently prepared. The EB content in the samples was determined based on the standard curve.

### Hematoxylin and eosin staining

On the 14th day post TBI, the anesthetized rats underwent the following procedures: First, physiological saline was perfused through the left ventricle, and the right atrium was incised to completely clear the blood from the brain tissue. The brain tissue was then extracted and fixed in 4% paraformaldehyde at room temperature for 24 hours. Subsequently, a gradient dehydration process using 70%, 80%, 90%, 95%, and 100% ethanol was performed, followed by paraffin embedding and sectioning. Finally, the brain tissue sections were stained with hematoxylin and eosin. In summary, our procedures involved deparaffinizing the sections and rehydrating them using gradient ethanol, staining with hematoxylin for 3–5 minutes, followed by differentiation and bluing, and then staining with eosin for 5 minutes. Afterward, the sections were dehydrated, cleared, and cover slipped. Ultimately, the sections were examined using an optical microscope (Nikon Eclipse C1; Nikon, Tokyo, Japan), and images were captured and analyzed.

### Fluoro-Jade B staining

Fluoro-Jade B (FJB) staining was utilized to evaluate neuronal death. The deparaffinization and rehydration steps for the sections were as follows: The sections were sequentially immersed in environmentally friendly deparaffinizing agent (G1128) I for 10 minutes, environmentally friendly deparaffinizing agent II for 10 minutes, environmentally friendly deparaffinizing agent III for 10 minutes, followed by 5 minutes each in absolute ethanol I, absolute ethanol II, and absolute ethanol III, and then washed with distilled water. Subsequently, the sections were incubated with FJB working solution (1:400, Merck Life Science AG310) overnight at 4°C. After staining the nuclei with DAPI for 8 minutes, the sections were rinsed with pure water, air-dried, and then briefly treated with xylene for 1 minute before mounting with ultrapure quick-drying mounting medium (G1404-100 mL). The number of FJB+ cells was quantified under a microscope (Nikon Eclipse C1; Nikon, Japan) using NIH ImageJ Software (Bethesda, MD, USA) by a technician who was blinded to the experimental design. At least three slides per rat sample were stained and analyzed for cell counting.

### TUNEL staining and Nissl staining

Based on the manufacturer’s instructions, we employed the TUNEL assay kit (G1502, Servicebio, China) for labeling paraffin-embedded sections in order to identify apoptotic cells in the cortical tissue. The TUNEL kit was marked with CY3 fluorescein, resulting in the visualization of red-stained positive apoptotic cell nuclei, while DAPI was employed to stain regular cell nuclei, which appeared in blue. The apoptotic index was determined as the ratio of TUNEL-positive nuclei to the total number of nuclei. The quantification of TUNEL + cells was carried out using a microscope (Nikon Eclipse C1; Nikon, Japan) and analyzed with NIH ImageJ Software (Bethesda, MD, USA) by a technician who was unaware of the experimental design. For each Rat sample, a minimum of three slides were stained and evaluated for cell counting. For Nissl staining, the sections were immersed in Nissl staining solution (G1036, Servicebio) for 4 minutes, followed by rinsing with double-distilled water. Subsequently, a slight differentiation was achieved using 0.1% glacial acetic acid. After thorough water washing, the sections were air-dried in an oven, dehydrated with xylene until transparent (approximately 10 minutes), and finally mounted with neutral gum. Photomicrographs were acquired using a light microscope (Nikon Eclipse C1; Nikon, Japan).

### Immunofluorescence staining

Following dewaxing and hydration procedures, brain tissue sections undergo antigen retrieval. Upon completion of the repair process, the sections are initially treated with a 3% BSA solution, and this sealing step lasts for 30 minutes. Subsequently, a pre-prepared primary antibody solution (CD31, Abcam, AB281583, 1:500; Brdu, Abcam, Ab6326, 1:200; OX1R, Huabio, China, ER1914-44, 1:200) is carefully applied dropwise onto the sections. The sections are then placed flat within a humid chamber and allowed to incubate overnight at 4°C. On the following day, corresponding secondary antibody solutions (Cy3-conjugated anti-goat IgG against rabbit) are added dropwise. The sections are kept in a light-protected environment and incubated at room temperature for 50 minutes. Afterward, DAPI staining solution is added dropwise, and the sections continue to be incubated at room temperature under light-protected conditions for an additional 10 minutes. Following the staining process, an autofluorescence quenching reagent is applied for 5 minutes. Subsequently, the sections are rinsed with running water for 10 minutes. Lastly, the sections are sealed using an anti-fading mounting medium designed for fluorescence quenching. Image acquisition is performed using a fluorescence microscope (Nikon Eclipse C1; Nikon, Japan).

### Western blotting

After the completion of behavioral assessments in the second week following TBI in rats, cardiac perfusion was performed for euthanasia. Subsequently, damaged regions of the brain cortex were collected for protein analysis. Protein extraction followed the manufacturer’s provided protocols, utilizing the BCA protein quantification kit (Solarbio, PC0020) to determine protein concentration. Equal amounts of protein were separated through polyacrylamide gel electrophoresis and then swiftly transferred to a nitrocellulose (NC) membrane using rapid transfer buffer. Next, a 2-hour room temperature blocking step was carried out using 5% non-fat milk. Subsequently, incubation was performed at 4°C overnight using anti-CD31 (Abcam, AB281583, 1:1000), anti-VEGFA (Abcam, AB214424, 1:1000), anti-Orexin-A (bs-15509R, 1:1000), anti-OX1R (Huabio, ER1914-44, 1:1000), anti-TF (Immunoway, USA, YT5194, 1:1000), anti-p-TF (Immunoway, YP0733, 1:1000), anti-AKT (CST, USA, 4691, 1:1000), anti-p-AKT (CST, 4060, 1:2000), anti-ERK (CST, 4695, 1:1000), anti-p-ERK (CST, 4370, 1:2000), anti-GAPDH (Proteintech, China, 10494-1-AP1, 1:5000), anti-β-Tubulin (Immunoway, YT4780, 1:1000). Following antibody incubation, the membranes were washed three times with TBST solution, each wash lasting 15 minutes, followed by a 1-hour incubation at room temperature with corresponding HRP-conjugated secondary antibodies (ZSGB-BIO, China, ZB-2301, 1:10000). Finally, enhanced chemiluminescence (ECL) was employed to detect protein bands, and ImageJ software was utilized to measure the protein band’s optical density.

### Quantitative real-time polymerase chain reaction (qRT-PCR)

We isolated total RNA from traumatic brain tissue using Trizol reagent (Invitrogen, USA) with a sample size of (*n* = 3 per group). Following this, the extracted RNA was reverse transcribed using a commercially accessible Takara kit to produce complementary DNA (cDNA). Following the manufacturer’s instructions, we conducted quantitative real-time PCR (qRT-PCR) using SYBR Green PCR Master Mix (TransGen Biotech, China) and performed the PCR on a CFX96 real-time PCR detection system. The amplification protocol consisted of an initial denaturation step at 95°C for 30 seconds, followed by 40 cycles comprising denaturation at 95°C for 10 seconds, annealing at 60°C for 30 seconds, and extension at 60°C for 30 seconds. To standardize the mRNA expression levels of the target genes, we used GAPDH as the reference gene and analyzed the data utilizing the 2^−ΔΔCT^ method. The oligonucleotide PCR primers were procured from Sangon Biotech (Shanghai, China), and comprehensive information can be located in Table 1.

**Table 1:** Primers’ sequences used for real-time PCR analysis.

**Gene**	**Accession number**	**Sequence 5′–3′**	**Amplification length (bq)**
OXRI	NM_013064.2	Forward primer: GAGTGTTTGGGATGTITCGC	143
		Reverse primer: GAACTGCTCCCGAAATTTGC	
CD31	NM_031591.1	Forward primer: CTGGAGAAACCTGCCAAGTATG	138
		Reverse primer: GGTGGAAGAATGGGAGTTGCT	
VEGFA	NM_031836	Forward primer: ATCGGCAAAGTGGTCAAGAGAA	168
		Reverse primer: TAGGAGGCGGTAAGTGATGGG	
VEGFR1	NM_001309381.1	Forward primer: CAATGATGAAGCCCTGGAGTG	103
		Reverse primer: GCTCATCTCTCCTATGTGCTGG	
R-GAPDH	NM_017008.4	Forward primer: GATACCTGCCTAAATCCACCTCG	240
Reverse primer: TGTCCCACGCTATTCTTTGCC			

### Cell culture and drug administration

HUVEC cells (HUM-iCELL-e005) were procured from iCell Bioscience Inc. (China) and cultivated using specialized primary endothelial cell culture medium (PriMED-iCELL-002). These cells were cultured in a chamber with 5% CO_2_ and 95% air at a temperature of 37°C. In order to investigate the influence of Orexin-A (MedChemExpress, USA, HY-106224) on TNF-α (Abbkine, USA, PRP1013)-induced HUVEC cells and explore potential mechanisms, we subjected the cells to varying concentrations of Orexin-A (0.1, 0.5, and 1 μM), TNF-α (10 ng/mL), and the inhibitor LY3214996 (MedChemExpress, HY-101494) of the ERK1/2 signal transduction pathway (1 μM).

### Wound healing assay

To evaluate cell migration, we performed a scratch wound healing experiment, which involved the following specific procedures. Initially, primary HUVEC cells were seeded into 6-well culture plates (Corning Inc, Corning, NY, USA) and cultivated in specialized medium until reaching confluence. Subsequently, a single-cell layer was scratched using a sterile plastic pipette tip (20 μL), followed by three washes with PBS to eliminate any dislodged cells. The cells were then cultured for 24 hours in culture medium containing Orexin-A, TNF-α, LY3214996, or combinations thereof, supplemented with 1% fetal bovine serum (FBS). During the incubation period, cell migration was observed at both 0 and 24 hours using an optical microscope in three randomly selected fields. To calculate the percentage of gap closure, the following formula was employed: Gap closure percentage = (W0-W24)/W0 × 100%, where W0 represents the initial scratch gap width at 0 hours, and W24 represents the scratch gap width at 24 hours.

### Tube formation assay

To explore the potential beneficial effects of Orexin-A on vascular formation capacity in TNF-α injured HUVEC cells and its possible association with the ERK1/2 pathway, we conducted *in vitro* tube formation assays. The specific steps were as follows. Initially, we added 150 μL of Matrigel (BD, Corning Matrigel, 354262) diluted to a concentration of 6 mg/ml into each well of a 48-well culture plate (Corning Inc., Corning, NY, USA) and allowed it to solidify overnight at 37°C. Subsequently, HUVEC cells were seeded into the 48-well plate (60000 cells per well) containing 300 μL of medium (without FBS). The culture medium in each well contained varying concentrations of Orexin-A (0.1, 0.5, and 1 μM), TNF-α (10 ng/mL), LY3214996 (1 μM), or different combinations thereof. Afterward, the culture plates were incubated at 37°C for 6 hours. The resulting tubular structures were observed using an optical microscope and analyzed using ImageJ Software (Bethesda, MD, USA).

### *In vivo* rat Matrigel plug assay

To further explore the influence of Orexin-A on vascular formation capacity in TNF-α-injured HUVEC cells and its potential relationship with the ERK1/2 pathway, we conducted *in vivo* Matrigel plug experiments. The procedures were as follows: HUVEC cell suspensions, containing Orexin-A, TNF-α, LY3214996, or various combinations thereof, were prepared and thoroughly mixed with Matrigel (BD, Corning Matrigel, 354262) to achieve a final Matrigel concentration of 7 mg/ml. These mixtures were then subcutaneously injected into the axillary region of rats. After 7 days, the rats were euthanized, and Matrigel plugs were retrieved, preserved using 4% paraformaldehyde, embedded in paraffin wax, and subsequently sectioned. Each plug was subjected to hematoxylin and eosin staining and CD31 immunofluorescence staining. Quantitative assessment of the fluorescence intensity was performed using the ImageJ software, allowing for the evaluation of vascular formation.

### Statistical analysis

Data that conform to a normal distribution is displayed as mean ± standard deviation. To identify significant distinctions among the groups in this dataset, we applied one-way analysis of variance (ANOVA) followed by Tukey’s post hoc analysis. In cases where the data deviates from a normal distribution, the Kruskal-Wallis test is employed to evaluate significant differences between groups. We conducted statistical analysis of the data using GraphPad Prism 8.0 software, and a significance threshold of *P* < 0.05 was considered indicative of statistical significance.

### Data availability statement

Data utilized for supporting research discoveries remain obtainable from corresponding author as required.

## RESULTS

### tDCS promotes angiogenesis at the injury site and reduces neurological deficits and tissue damage

**Figure 1:**
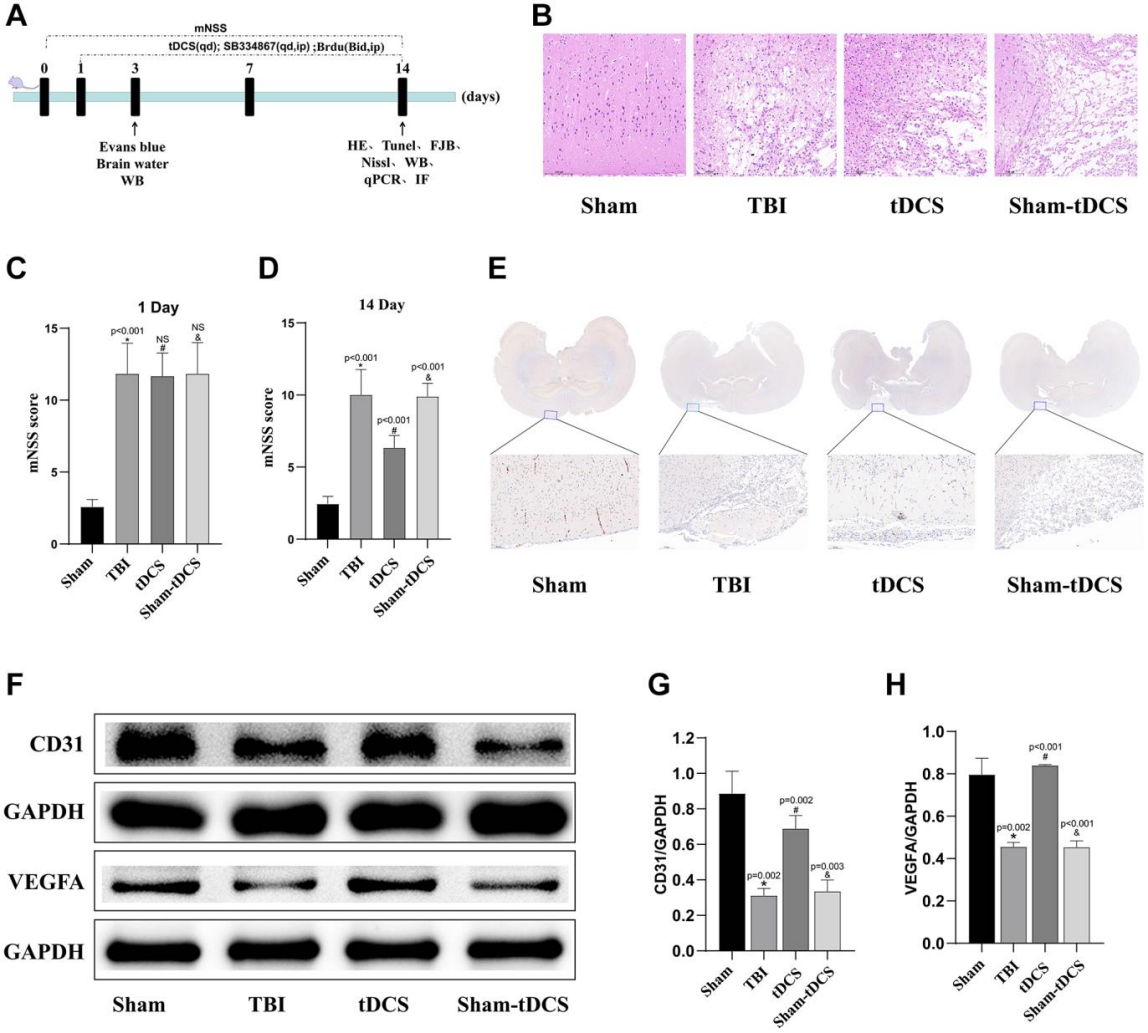
**tDCS promotes angiogenesis at the injury site and reduces neurological deficits and tissue damage.** (**A**) Timeline of the animal experiments. (**B**) Representative gross images of brain tissue from the four groups of rats and hematoxylin and eosin staining were used to visualize the necrotic areas (*n* = 3). (**C**, **D**) After TBI, we assessed the neural function of rats using the Modified Neurological Severity Scores (mNSS) before and two weeks following tDCS treatment (*n* = 6). (**E**) Immunohistochemistry staining for the representative vascular marker CD31 was performed on brain tissue from the four groups of rats at the injury site (*n* = 3). (**F**) Representative western blotting images of CD31 and VEGFA on 14 days post-TBI. Using GAPDH as an internal reference for band density normalization (*n* = 3). (**G**, **H**) Quantification of western blotting for CD31, VEGFA expression (*n* = 3). Results are expressed as means ± standard deviation, the statistical significance of differences was evaluated by One-way ANOVA, ^*^represents the comparison between the Sham group and the TBI group, ^#^signifies the comparison between the TBI group and the tDCS group, and ^&^denotes the comparison between the tDCS group and the Sham-tDCS group. *P* < 0.05 indicates statistical significance.

The schematic diagram illustrating the animal experimental procedure is depicted in Figure 1A. To assess the therapeutic efficacy of tDCS in TBI, we conducted hematoxylin and eosin (HE) staining on brain tissue samples from each group. The results revealed that the brain tissue structure in the sham operation group appeared clear and dense, with normal and evenly arranged neuronal structures. Conversely, both the TBI group and sham-tDCS group exhibited sparse tissue structures characterized by widespread vacuolar changes and cell death. Notably, tDCS treatment significantly ameliorated brain tissue lesions and reduced cell death (Figure 1B). Neurological deficit assessment using mNSS was conducted on TBI rats on the first day and second week post-injury. On the first day post-TBI, prior to tDCS treatment, the mNSS scores in the TBI group markedly increased compared to the sham group (*p* < 0.001), while no significant differences were observed among the treatment groups (*p* > 0.05). However, by the two-week mark after tDCS treatment, the TBI group exhibited higher scores compared to the sham group (*p* < 0.001). Importantly, tDCS treatment significantly reduced mNSS scores (*p* < 0.001), whereas the sham-tDCS group did not show a reduction in scores (*p* < 0.001), indicating the neuroprotective effect of tDCS against neural injury (1D: F (3, 20) = 108.2, *p* < 0.001; 2W: F (3, 20) = 60.48, *p* < 0.001; Figure 1C, 1D). Immunohistochemical staining analysis of brain tissue sections revealed a substantial decrease in the number of blood vessels at the injury site within the TBI group. However, following tDCS treatment, a significant increase in blood vessel count was observed compared to the TBI group. In contrast, brain tissue sections from the sham-tDCS group showed no noticeable increase in blood vessel count (Figure 1E). Furthermore, we investigated protein expression in the injured brain tissue using western blotting (Figure 1F). The results indicated a significant decrease in the expression levels of CD31 and VEGFA proteins in the TBI group compared to the Sham group (*p* < 0.05). Conversely, following tDCS treatment, a marked increase in the expression levels of CD31 and VEGFA proteins was observed relative to the TBI group (*p* < 0.05). However, in the sham-tDCS group, no discernible elevation in CD31 and VEGFA protein expression levels was noted (CD31: F (3, 8) = 34.64, *p* < 0.001; VEGFA: F (3, 8) = 71.35, *p* < 0.001; Figure 1G, 1H). These findings collectively highlight the beneficial impact of tDCS on the repair of brain injuries following TBI, particularly its role in promoting angiogenesis.

### tDCS promotes the expression of OXA and OX1R in TBI rats

**Figure 2:**
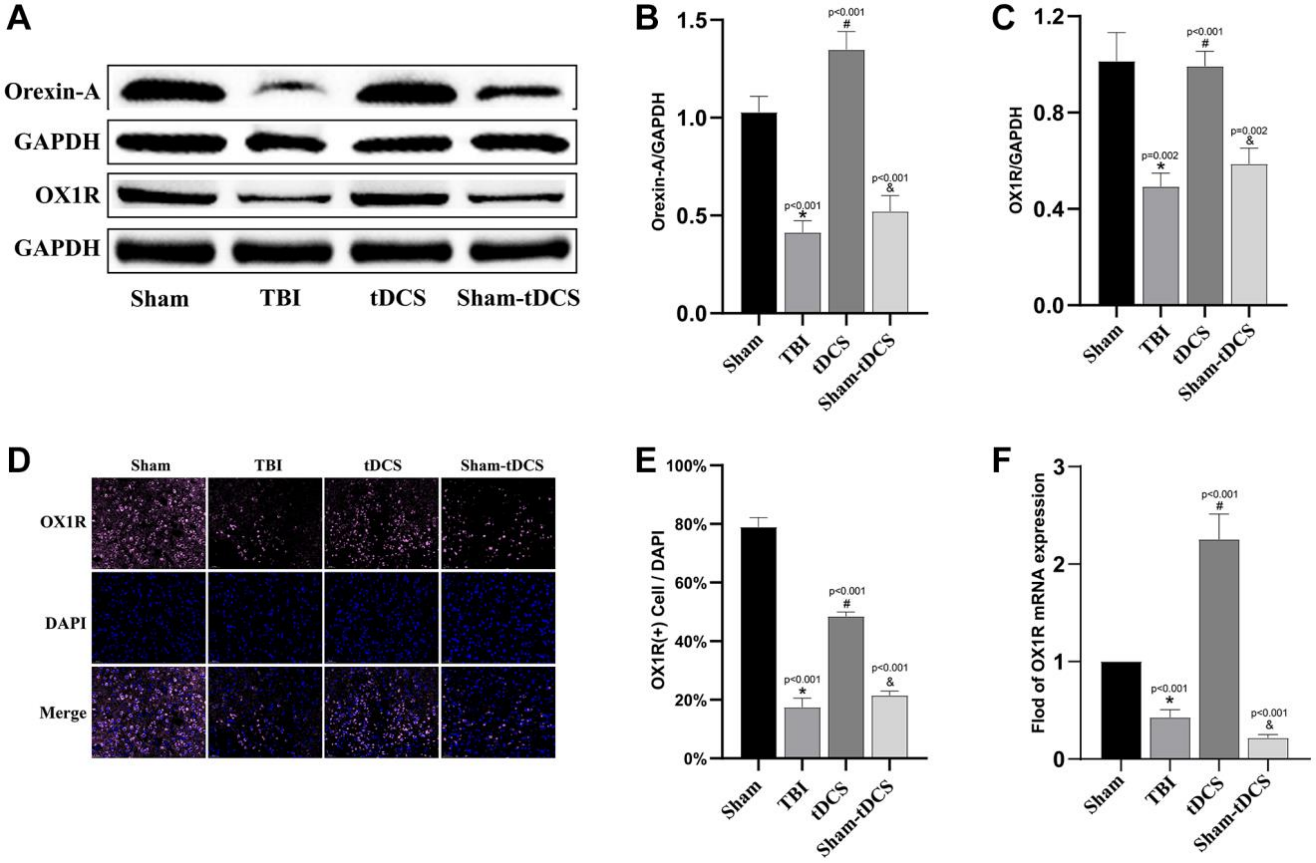
**tDCS promotes the expression of OXA and OX1R in TBI rats.** (**A**) Representative western blotting images of Orexin-A and Orexin-A receptor 1 (OX1R) on 14 days post-TBI. Using GAPDH as an internal reference for band density normalization (*n* = 3). (**B**) Quantification of western blotting for Orexin-A expression (*n* = 3). (**C**) Quantification of western blotting for OX1R expression (*n* = 3). (**D**) Representative immunofluorescence images of OX1R expression in brain tissue (*n* = 3). (**E**) Quantification of immunofluorescence for OX1R expression (*n* = 3). (**F**) Quantitative analysis of mRNA levels of OX1R expression (*n* = 3). Results are expressed as means ± standard deviation, the statistical significance of differences was evaluated by One-way ANOVA, ^*^represents the comparison between the Sham group and the TBI group, ^#^signifies the comparison between the TBI group and the tDCS group, and ^&^denotes the comparison between the tDCS group and the Sham-tDCS group. *P* < 0.05 indicates statistical significance.

Based on previous research, OXA has been identified as a crucial regulator of angiogenesis [12, 26]; Our preliminary investigations have shown that OXA plays a crucial role in brain injury repair [27]. Consequently, we postulate that tDCS may facilitate angiogenesis by upregulating OXA expression. To substantiate this hypothesis, we conducted the following experiments: employing western blotting to assess OXA and OX1R protein levels (Figure 2A). The findings unveiled a significant reduction in OXA and OX1R expression in the TBI group compared to the sham group (*p* < 0.05), whereas the tDCS group exhibited a notable increase in OXA and OX1R expression (*p* < 0.001). Additionally, the Sham-tDCS group displayed a marked decrease in OXA and OX1R expression relative to the tDCS group (*p* < 0.05) (OXA: F (3, 8) = 91.79, *p* < 0.001; OX1R: F (3, 8) = 34.50, *p* < 0.001; Figure 2B, 2C). Furthermore, immunofluorescence analysis of rat brain slices demonstrated a significant decrease in the number of OX1R-positive cells in the TBI group compared to the sham group (*p* < 0.001), whereas the tDCS group exhibited a substantial increase in OX1R-positive cell count (*p* < 0.001), with no discernible elevation in the Sham-tDCS group (*p* < 0.001) (F (3, 8) = 393.1, *p* < 0.001, Figure 2D, 2E). Lastly, assessment of OX1R-associated mRNA expression in the injured brain tissue revealed a significant reduction in the TBI group compared to the sham group (*p* < 0.001). Conversely, the tDCS group displayed a marked upsurge in OX1R mRNA expression (*p* < 0.001), while OX1R mRNA expression significantly decreased in the Sham-tDCS group compared to the tDCS group (*p* < 0.001) (F (3, 8) = 133.2, *p* < 0.001, Figure 2F). In summary, tDCS exhibits the potential to enhance OXA and OX1R expression in the brain tissue of rats with TBI.

### The crucial role of OXA in tDCS reduced necrotic neuronal degeneration in TBI rats

**Figure 3:**
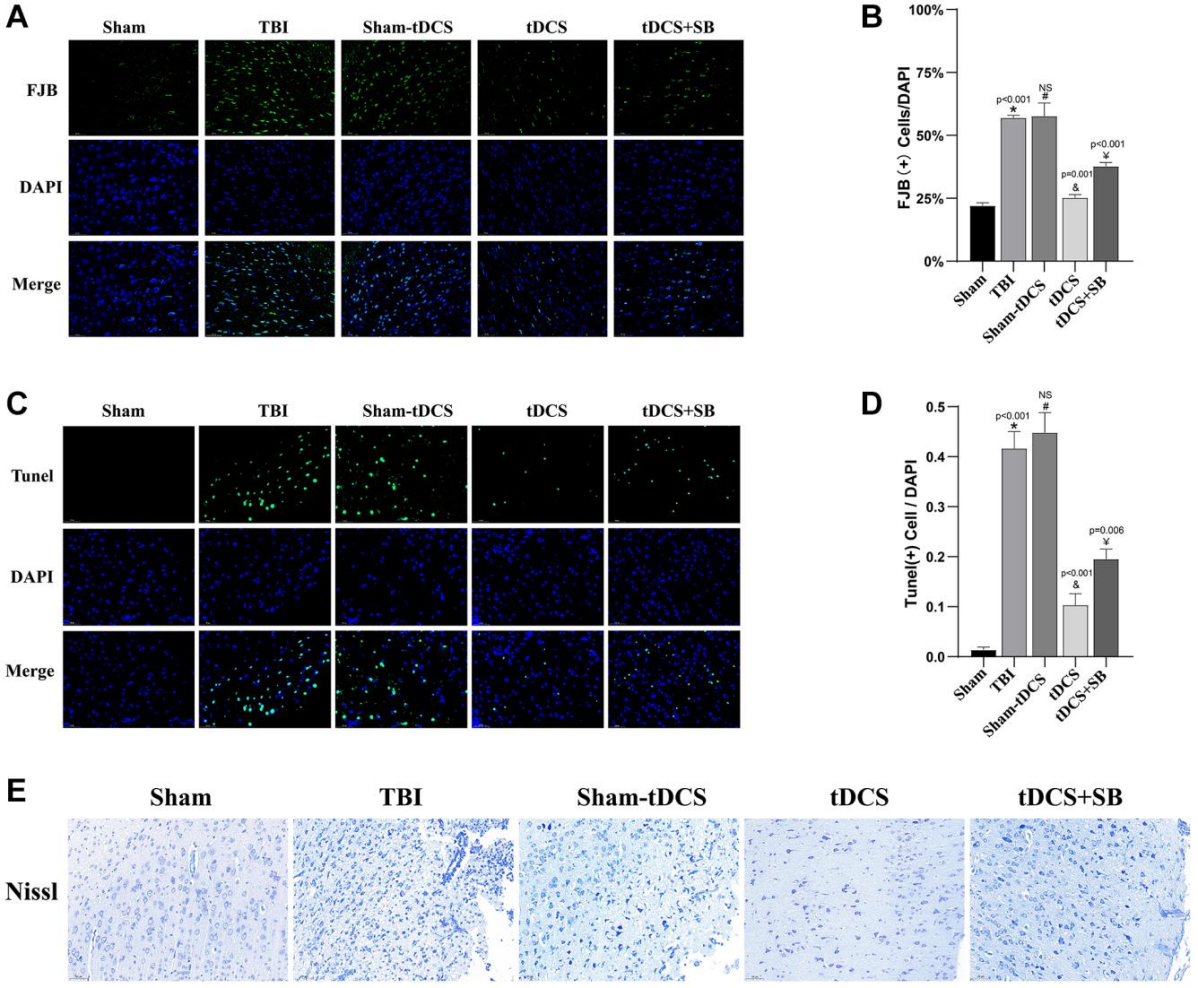
**The crucial role of OXA in tDCS reduced necrotic neuronal degeneration in in TBI rats.** (**A**) Representative images of Fluoro Jade B (FJB) staining of the injured peripheral cortex in 2W after injury (*n* = 3). (**B**) Quantitative analysis reveals the proportion of FJB-positive cells to DAPI (*n* = 3). (**C**) Representative images of Tunel staining of the injured peripheral cortex in 2W after injury (*n* = 3). (**D**) Quantitative analysis reveals the proportion of Tunel-positive cells to DAPI (*n* = 3). (**E**) Representative images of Nissl staining of the injured peripheral cortex in 2W after injury (*n* = 3). Results are expressed as means ± standard deviation, the statistical significance of differences was evaluated by One-way ANOVA, ^*^denotes the comparison between the Sham group and the TBI group, ^#^represents the comparison between the TBI and the Sham-tDCS group, ^&^signifies the comparison between the tDCS group and the Sham-tDCS group, and ^¥^indicates the comparison between the tDCS group and the tDCS+SB334867 group. *P* < 0.05 indicates statistical significance.

To investigate the impact of tDCS treatment on neuronal degeneration and necrosis following TBI, we conducted Fluoro-Jade B (FJB) staining during the second week post-injury (F (4, 10) = 78.93, *p* < 0.001; Figure 3A, 3B). The results revealed a significant increase in FJB (+) cells in both the TBI group and the sham-tDCS group compared to the sham group (*p* < 0.05). However, following tDCS treatment, there was a substantial reduction in FJB (+) cells (*p* = 0.001). Nevertheless, SB334867 significantly reversed this effect (*p* < 0.05). Furthermore, we utilized Terminal deoxynucleotidyl transferase dUTP nick end labeling (TUNEL) staining to assess cell apoptosis (F (4, 10) = 148, *p* < 0.001; Figure 3C, 3D) and found a higher number of TUNEL (+) cells in both the TBI group and the sham-tDCS group (*p* < 0.001). Nevertheless, tDCS treatment resulted in a significant reduction in TUNEL (+) cell count (*p* < 0.001). However, SB334867 significantly reversed this effect (*p* = 0.006). Additionally, Nissl staining was conducted to further observe neuronal damage (Figure 3E), revealing cytoplasmic shrinkage and nuclear condensation in the TBI group and the sham-tDCS group. However, tDCS treatment contributed to alleviating these histopathological changes. Similarly, SB334867 significantly reversed this effect (Figure 3E). Collectively, these findings indicate that tDCS treatment effectively attenuates TBI-induced ipsilateral cortical neuronal damage and is closely related to Orexin-A.

### The crucial role of OXA in tDCS promotes angiogenesis at the site of injury after TBI

**Figure 4:**
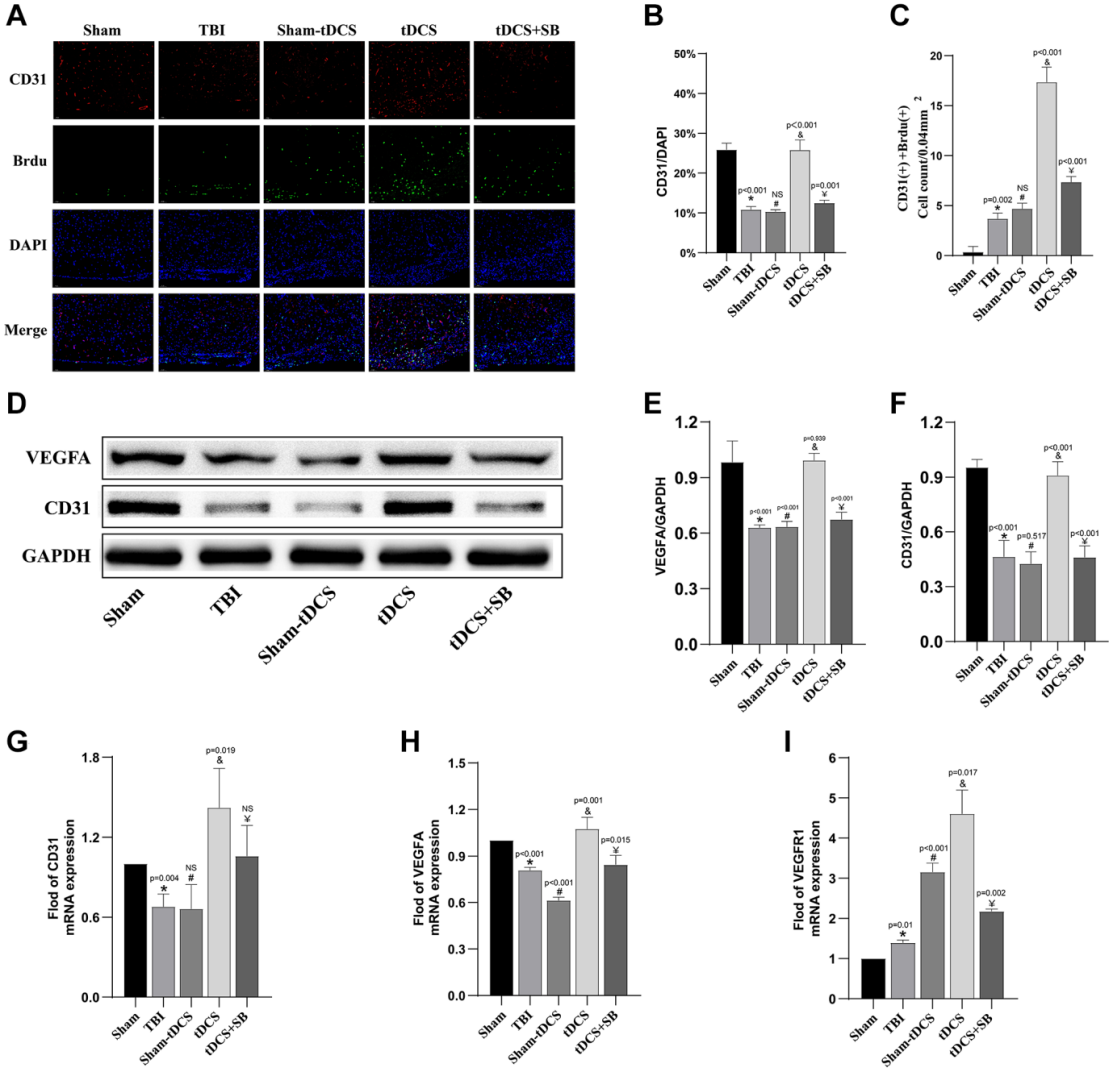
**The crucial role of OXA in tDCS promotes angiogenesis at the site of injury after TBI.** (**A**) Representative immunofluorescence images of CD31+ Brdu-positive cell in brain tissue in 2W after injury (*n* = 3). (**B**) Quantitative analysis reveals the proportion of CD31-positive cells (*n* = 3). (**C**) Quantitative analysis reveals the count of CD31+ Brdu-positive cells (*n* = 3). (**D**) Representative western blotting images of CD31 and VEGFA on 14 days post-TBI. Using GAPDH as an internal reference for band density normalization (*n* = 3). (**E**) Quantification of western blotting for VEGFA expression (*n* = 3). (**F**) Quantification of western blotting for CD31 expression (*n* = 3). (**G**) Quantitative analysis of mRNA levels of CD31 expression (*n* = 3). (**H**) Quantitative analysis of mRNA levels of VEGFA expression (*n* = 3). (**I**) Quantitative analysis of mRNA levels of VEGFR1 expression (*n* = 3). Results are expressed as means ± standard deviation, the statistical significance of differences was evaluated by One-way ANOVA, ^*^denotes the comparison between the Sham group and the TBI group, ^#^represents the comparison between the TBI and the Sham-tDCS group, ^&^signifies the comparison between the tDCS group and the Sham-tDCS group, and ^¥^indicates the comparison between the tDCS group and the tDCS+SB334867 group. *P* < 0.05 indicates statistical significance.

To further investigate the impact of tDCS on brain vasculature in the injury site after TBI, we conducted CD31+Brdu immunofluorescence staining two weeks after the brain injury. CD31 serves as an endothelial marker for identifying brain microvessels after TBI [28], while BrdU staining labels dividing cells, and their co-localization serves as a marker for neovascularization. Immunofluorescence images were collected around the injury boundary region in the ipsilateral hemisphere (CD31: F (4, 10) = 91.43, *p* < 0.001; CD31+BrdU: F (4, 10) = 171.1, *p* < 0.001; Figure 4A–4C). Compared to the sham group, both the TBI group and the sham-tDCS group exhibited a significant reduction in the proportion of CD31-positive cells (*p* < 0.001); however, in the tDCS treatment group, the number of CD31-positive cells was significantly increased (*p* < 0.001). However, SB334867 significantly reversed this effect (*p* = 0.001). These findings indicate that tDCS treatment significantly enhances the number of microvessels at the brain injury site in TBI rats. We further investigated the quantity of BrdU+/CD31+ cells and found a significant increase in the tDCS group compared to both the TBI group and the sham-tDCS group (*p* < 0.001). Similarly, SB334867 significantly reversed this effect (*p* < 0.001). To explore the potential effects of tDCS on angiogenesis and neuroprotection, we conducted western blot analysis of VEGFA and CD31 levels in the brain tissue surrounding the injury site (VEGFA: F (4, 10) = 30.88, *p* < 0.001; CD31: F (4, 10) = 43.42, *p* < 0.001; Figure 4D–4F). Compared to the sham group, the TBI group exhibited decreased levels of VEGFA (*p* < 0.001) and CD31 (*p* < 0.001) proteins. tDCS treatment restored the expression of VEGFA (*p* < 0.001) and CD31 (*p* = 0.001) proteins; however, this effect was significantly suppressed by SB334867 (*p* < 0.05). We also extracted RNA from the cortex around the injury site and performed RT-PCR to analyze angiogenesis-related factors (VEGFA: F (4, 10) = 46.75; CD31: F (4, 10) = 8.011; VEGFR1: F (4, 10) = 75.69; Figure 4G–4I). Compared to the sham group, TBI rats showed significant reductions in angiogenesis-related factors VEGFA (*p* < 0.001) and CD31 (*p* = 0.004), but tDCS treatment significantly upregulated VEGFA (*p* = 0.001) and CD31 (*p* = 0.019). However, SB334867 significantly inhibited the effect of tDCS on upregulating VEGFA (*p* < 0.05). Although there was no statistically significant difference for CD31, there was a decrease in overall expression levels. Regarding VEGFR1, compared to the sham group, both the TBI group and sham-tDCS group exhibited increases (*p* < 0.05), with tDCS treatment resulting in a more pronounced increase (*p* < 0.05). Similarly, SB334867 significantly reversed this effect (*p* < 0.05). In summary, tDCS can promote neovascularization of brain tissue around the injury boundary zone after TBI, with Orexin-A playing a key role.

### The crucial role of OXA in tDCS-mediated restoration of the blood-brain barrier in TBI rats

**Figure 5:**
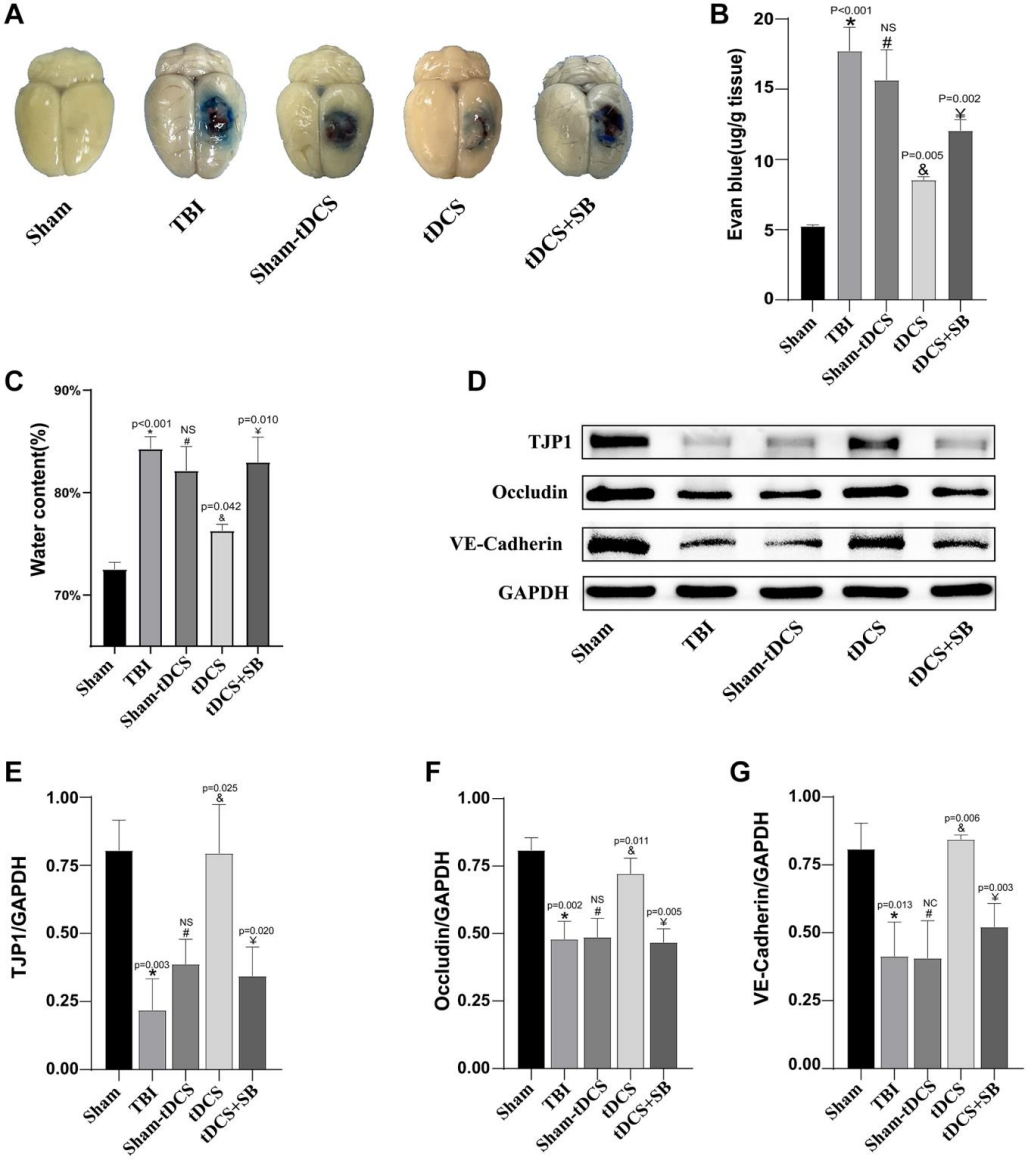
**The crucial role of OXA in tDCS-mediated restoration of the blood-brain barrier in TBI rats.** (**A**) On the third day following TBI, representative images of Evans blue extravasation in the brains of each experimental group were obtained (*n* = 3). (**B**) Quantitative analysis of the EB dye extravasation (*n* = 3). (**C**) Brain hemisphere water content three days after TBI (*n* = 3). (**D**) Representative western blotting images of TJP1, Occludin and VE-Cadherin on 14 days post-TBI. Using GAPDH as an internal reference for band density normalization (*n* = 3). (**E**) Quantification of western blotting for TJP1 expression (*n* = 3). (**F**) Quantification of western blotting for Occludin expression (*n* = 3). (**G**) Quantification of western blotting for VE-Cadherin expression (*n* = 3). Results are expressed as means ± standard deviation, the statistical significance of differences was evaluated by One-way ANOVA, ^*^denotes the comparison between the Sham group and the TBI group, ^#^represents the comparison between the TBI and the Sham-tDCS group, ^&^signifies the comparison between the tDCS group and the Sham-tDCS group, and ^¥^indicates the comparison between the tDCS group and the tDCS+SB334867 group. *P* < 0.05 indicates statistical significance.

We further assessed the protective effects of tDCS on blood-brain barrier (BBB) extravasation of Evans Blue dye and brain tissue hydration on the third day following TBI (F (4, 10) = 46.65, *p* < 0.001; Figure 5A, 5B). Our findings revealed a significant increase in intracerebral extravasation of Evans Blue dye in the TBI group compared to the sham group (*p* < 0.001), indicating exacerbated BBB permeability due to TBI. Notably, sham tDCS failed to alter the post-traumatic intracerebral leakage of Evans Blue dye (*p* > 0.05). In contrast, tDCS treatment significantly reduced the leakage of Evans Blue dye (*p* = 0.005). However, it’s important to note that SB334867 significantly attenuated the therapeutic efficacy of tDCS (*p* = 0.002). Additionally, we investigated the effects of tDCS on post-TBI brain edema (F (4, 10) = 27.06, *p* < 0.001; Figure 5C). The TBI group exhibited significantly higher brain tissue hydration levels compared to the sham group (*p* < 0.001). While sham tDCS did not ameliorate post-traumatic brain edema (*p* > 0.05), tDCS treatment led to a notable improvement in brain edema (*p* = 0.042). Nevertheless, similar to previous observations, SB334867 once again compromised the effectiveness of tDCS treatment (*p* = 0.010). Taken together, the cumulative results emphasize that tDCS treatment is capable of ameliorating the disruption of the blood-brain barrier and alleviating brain edema following TBI in rats. Tight junction (TJ) and adherens junction (AJ) proteins play pivotal roles in maintaining the integrity of the blood-brain barrier. In order to ascertain whether tDCS facilitates blood-brain barrier repair through the upregulation of TJ and AJ protein expression, we conducted western blotting analysis on the third day after TBI in rats (TJP1: F (4, 10) = 14.13, *p* = 0.0004; Occludin: F (4, 10) = 22.16, p < 0.001; VE-Cadherin: F (4, 10) = 12.99, *p* = 0.0006; Figure 5D–5G). The data revealed a significant decrease in the expression levels of TJP1 (*p* = 0.003), Occludin (*p* = 0.002), and VE-Cadherin (*p* = 0.013) in the TBI group. In contrast, sham tDCS exhibited no discernible impact on the expression of these proteins (*p* > 0.05). However, following tDCS treatment, there was a significant rescue of TJP1 (*p* = 0.025), Occludin (*p* = 0.011), and VE-Cadherin (*p* = 0.006) expression. Once again, SB334867 demonstrated its ability to compromise the effect of tDCS treatment (TJP1: *p* = 0.002, Occludin: *p* = 0.005, and VE-Cadherin: *p* = 0.003). In conclusion, our research outcomes suggest that tDCS treatment partially maintains the integrity of the blood-brain barrier post-TBI through the modulation of TJ and AJ protein expression, with an important role played by Orexin-A in this process.

### tDCS enhanced angiogenesis after TBI through OXA-TF-AKT/ERK pathway

**Figure 6:**
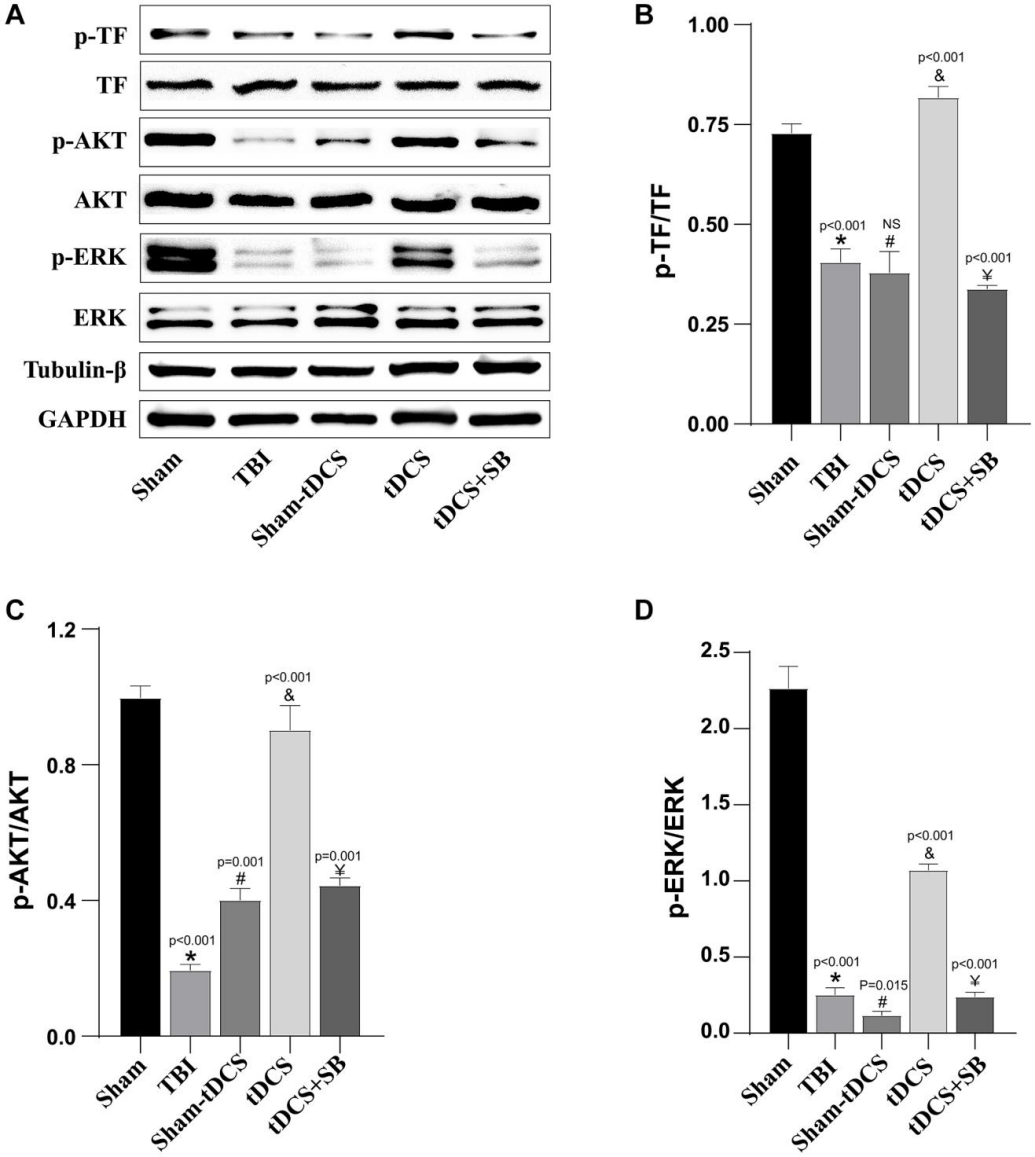
**tDCS enhanced angiogenesis after TBI through OXA-TF-AKT/ERK pathway.** (**A**) Representative western blotting images of TF, p-TF, AKT, p-AKT, ERK and p-ERK on 14 days post-TBI. Using GAPDH and β-Tubulin as internal reference for band density normalization (*n* = 3). (**B**) Quantification of western blotting for p-TF/TF expression (*n* = 3). (**C**) Quantification of western blotting for p-AKT/AKT expression (*n* = 3). (**D**) Quantification of western blotting for p-ERK/ERK expression (*n* = 3). Results are expressed as means ± standard deviation, the statistical significance of differences was evaluated by One-way ANOVA, ^*^denotes the comparison between the Sham group and the TBI group, ^#^represents the comparison between the TBI and the Sham-tDCS group, ^&^signifies the comparison between the tDCS group and the Sham-tDCS group, and ^¥^indicates the comparison between the tDCS group and the tDCS+SB334867 group. *P* < 0.05 indicates statistical significance.

Research has established a significant role for Orexin-A in promoting angiogenesis [12, 26], while simultaneously, the TF-AKT/ERK pathway has been identified as a crucial signaling pathway for regulating angiogenesis [29, 30]. This study reveals the capacity of tDCS to enhance the expression of Orexin-A, thus promoting angiogenesis. To further elucidate whether the tDCS-induced upregulation of Orexin-A affects the TF-AKT/ERK pathway, with the aim of facilitating post-TBI angiogenesis, we incorporated the Orexin-A inhibitor SB334867 into our investigation. Western blot techniques were employed to evaluate the expression of related proteins within the signaling pathways (p-TF: *p* < 0.001; p-AKT: F (4, 10) = 205, *p* < 0.001; p-ERK: F (4, 10) = 454.4, *p* < 0.001; Figure 6A–6D). The findings vividly illustrate that, compared to the sham-operated group, the expression levels of p-TF/TF (*p* < 0.001), p-AKT/AKT (*p* < 0.001), and p-ERK/ERK (*p* < 0.001) proteins notably decreased in the TBI group. However, following tDCS treatment, the expression levels of p-TF/TF (*p* < 0.001), p-AKT/AKT (*p* < 0.001), and p-ERK/ERK (*p* < 0.001) proteins were significantly upregulated, contrasting with the sham stimulation group which exhibited no corresponding changes, albeit with some fluctuations. Importantly, this upregulation was distinctly suppressed by the use of SB334867 (*p* < 0.05). In summary, our research outcomes indicate that tDCS enhances Orexin-A expression, thereby modulating the TF-AKT/ERK signaling pathway to facilitate post-TBI angiogene.

### Orexin-A improved scratch wound healing and angiogenesis of TNF-α injured HUVEC cells

**Figure 7:**
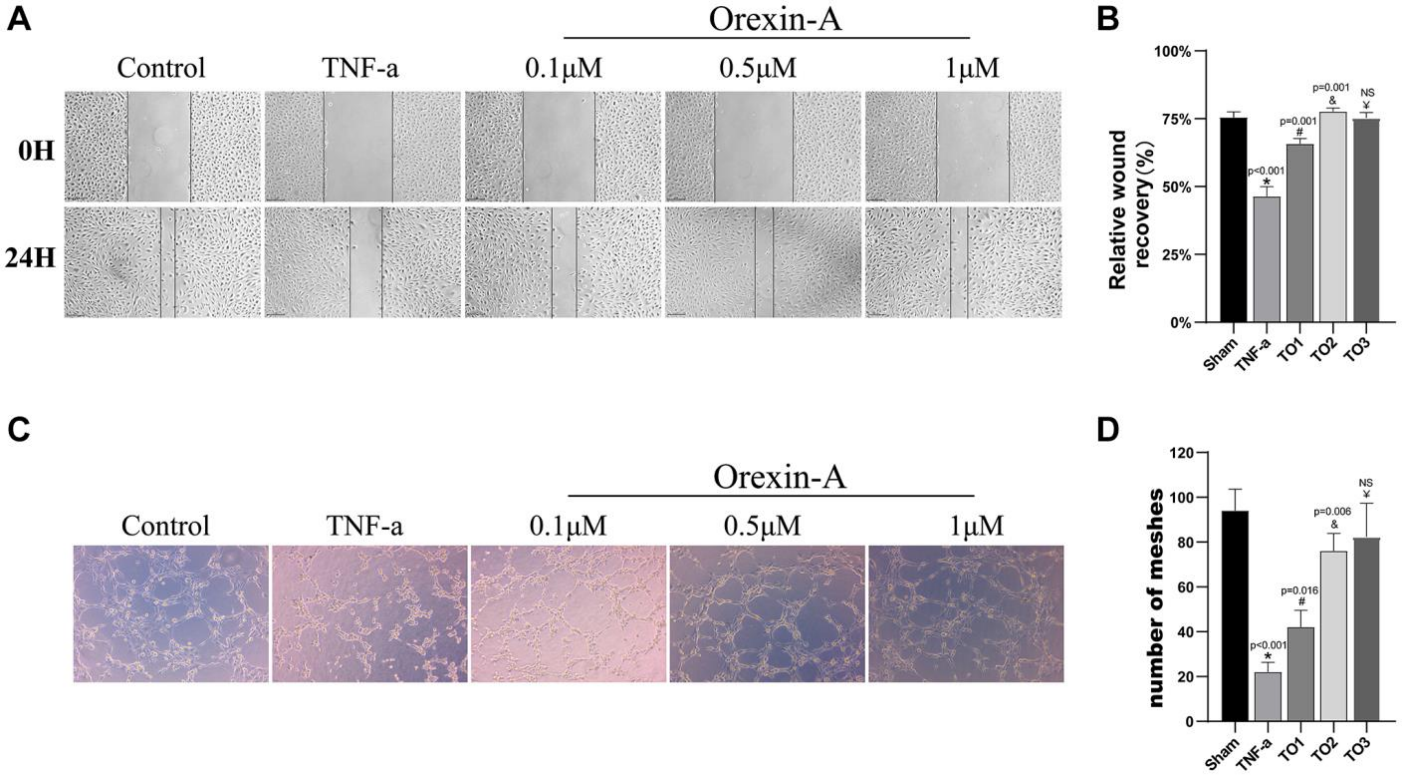
**Orexin-A improved scratch wound healing and angiogenesis of TNF-α-injured HUVEC cells.** (**A**) The image shows the effect of Orexin-A (TO1: 0.1 μM, TO2: 0.5 μM and TO3: 1 μM) on wound healing of TNF-α (10 ng/ml) injured cells. (**B**) Quantification of Orexin-A on wound closure (*n* = 3). (**C**) Representative images of the study effects of TNF-α (10 ng/ml) and the three concentrations (TO1: 0.1, TO2: 0.5 and TO3: 1 μM) of Orexin-A on tube formation. (**D**) Quantification of the effects of Orexin-A on the number of capillary-like tubes in TNF-α-treated cells (*n* = 3). Results are expressed as means ± standard deviation, the statistical significance of differences was evaluated by One-way ANOVA, ^*^denotes the comparison between the Sham group and the TBI group, ^#^represents the comparison between the TBI and the Sham-tDCS group, ^&^signifies the comparison between the tDCS group and the Sham-tDCS group, and ^¥^indicates the comparison between the tDCS group and the tDCS+SB334867 group. *P* < 0.05 indicates statistical significance.

To validate the potential role of Orexin-A in promoting *in vitro* angiogenesis, we further explored its effects on cell migration and capillary-like tube formation in TNF-α-induced HUVECs. Utilizing a scratch assay, we assessed the impact of Orexin-A on cell migration. The results (F (4, 10) = 93.52, *p* < 0.001; Figure 7A, 7B) demonstrated that TNF-α treatment led to a reduction in cell migratory capacity compared to the control group (*p* < 0.001). However, treatment with Orexin-A at concentrations of 0.1 μM (*p* = 0.001) and 0.5 μM (*p* = 0.001) significantly enhanced wound healing. Conversely, higher concentrations of Orexin-A (1 μM) did not show a proportional increase in reparative ability (p < 0.05). Furthermore, an *in vitro* capillary formation assay was conducted to assess the effect of Orexin-A on tube formation (F (4, 10) = 29.79, *p* < 0.001; Figure 7C, 7D). The results indicated that TNF-α treatment significantly inhibited HUVEC capillary formation compared to the control group (*p* < 0.001). Interestingly, Orexin-A at concentrations of 0.1 μM (*p* = 0.016) and 0.5 μM (*p* = 0.006) effectively restored the capillary-forming capacity of TNF-α-affected HUVECs. However, elevated levels of Orexin-A (1 μM) did not result in a more pronounced capillary-forming capability (*p* > 0.05). In summary, based on the above findings, we selected a concentration of 0.5 μM Orexin-A for further experiments to investigate its potential mechanisms of action.

### Orexin-A enhanced HUVEC angiogenesis and wound healing through TF-AKT/ERK pathway

**Figure 8:**
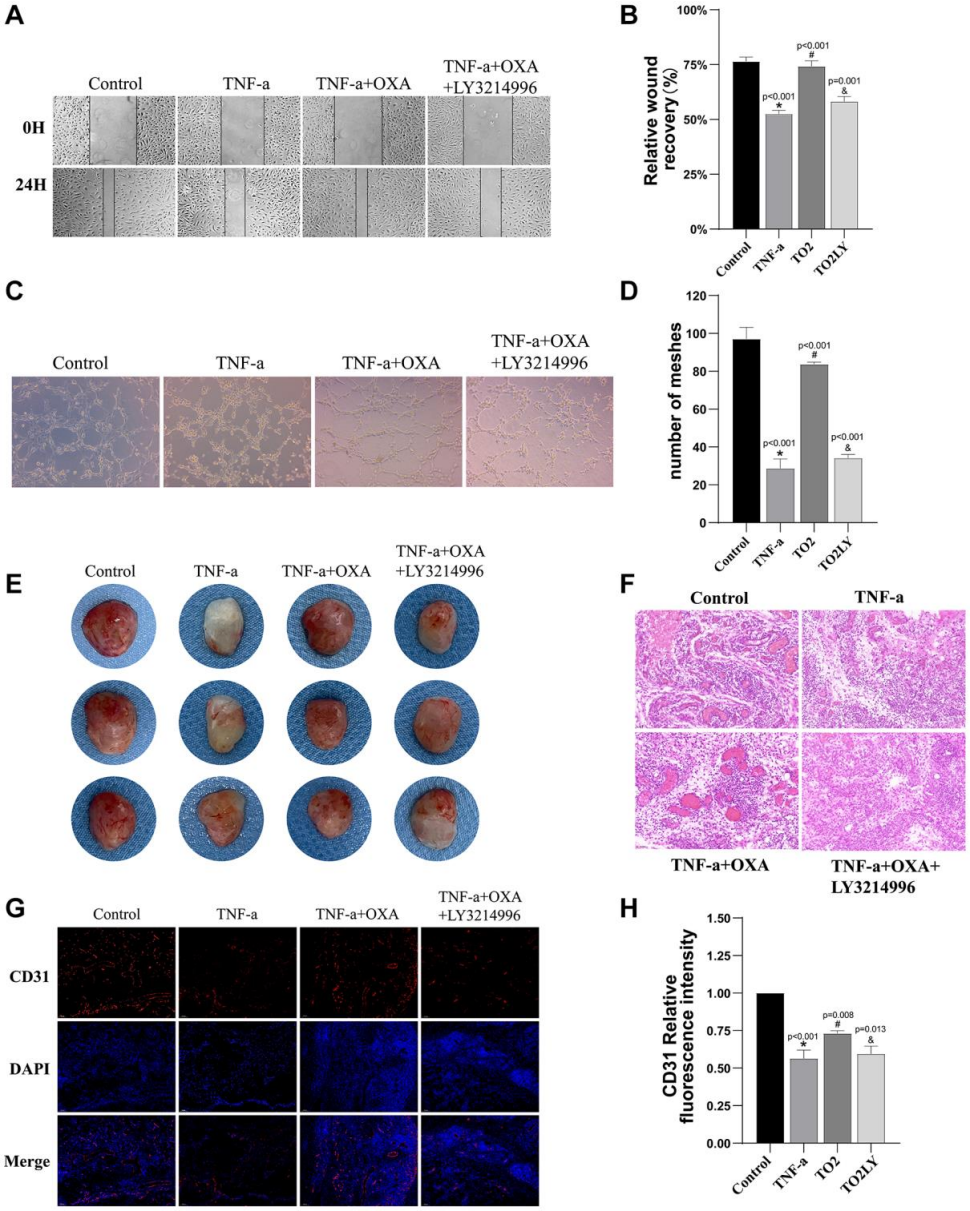
**Orexin-A enhanced HUVEC angiogenesis and wound healing through TF-AKT/ERK pathway.** (**A**) Representative images depicting the combined administration of LY3214996 (1 μM) and Orexin-A (0.5 μM) on wound healing effects. (**B**) The quantification of scratch assay results for each group is shown, measuring the closure of scratch gaps (*n* = 3). (**C**) Representative images illustrating the impact of the combined administration of LY3214996 (1 μM) and Orexin-A (0.5 μM) on vascular formation. (**D**) Displays the quantification of tube formation assay results for each group, calculating the number of formed tubes (*n* = 3). (**E**) Representative gross morphology of Matrigel *in situ* (*n* = 3). (**F**) Representative H&E-stained images of matrix plug sections (*n* =3). (**G**) Representative immunofluorescence images of CD31-positive cells within matrix plugs. (**H**) Quantitative analysis of the proportion of CD31-positive cells within matrix plugs (*n* = 3). Results are expressed as means ± standard deviation, the statistical significance of differences was evaluated by One-way ANOVA, ^*^denotes the comparison between the Sham group and the TBI group, ^#^represents the comparison between the TBI and the Sham-tDCS group, ^&^signifies the comparison between the tDCS group and the Sham-tDCS group, and ^¥^indicates the comparison between the tDCS group and the tDCS+SB334867 group. *P* < 0.05 indicates statistical significance.

To investigate whether the TF-AKT/ERK pathway mediates the scratch healing and angiogenic effects of Orexin-A on HUVEC cells, we utilized the ERK1/2 inhibitor LY3214996 (1 μM). The results revealed that Orexin-A significantly enhanced the migration of TNF-α-injured endothelial cells (*p* < 0.001). However, the combined treatment of LY3214996 and Orexin-A markedly reversed this effect (*p* = 0.001) (F (3, 8) = 91.36, *p* < 0.001, Figure 8A, 8B). Similarly, the angiogenic activity of TNF-α-injured cells, enhanced by Orexin-A, was significantly reversed by LY3214996 (*p* < 0.001) (F (3, 8) = 91.36, *p* < 0.001, Figure 8C, 8D). Furthermore, we investigated the impact of Orexin-A on vascular neogenesis using *in vivo* Matrigel plug experiments (Figure 8E, 8F). Our findings demonstrated that TNF-α significantly inhibited intravascular neogenesis, while Orexin-A effectively mitigated these inhibitory effects. However, co-administration of the ERK1/2 inhibitor LY3214996 counteracted the effects of Orexin-A (Figure 8E). Hematoxylin and eosin (HE) staining of Matrigel plugs revealed a substantial reduction in the number of vascular encirclements in the TNF-α group compared to the Sham group. Administration of Orexin-A resulted in a notable increase in vascular encirclements, which was significantly reduced upon concurrent use of the ERK1/2 inhibitor LY3214996 (Figure 8F). Further quantitative analysis through immunofluorescence demonstrated a significant reduction in the average fluorescence intensity of CD31-positive cells in the TNF-α group compared to the Sham group (*p* < 0.001). Conversely, treatment with Orexin-A led to a pronounced increase in the average fluorescence intensity of CD31-positive cells (*p* = 0.008). However, concurrent administration of the ERK1/2 inhibitor LY3214996 resulted in a significant reduction in the average fluorescence intensity of CD31-positive cells (*p* = 0.013) (F (3, 8) = 75.32, *p* < 0.001, Figure 8G, 8H). These findings highlight the potential of Orexin-A to enhance HUVEC cell migration and blood vessel formation, likely through involvement of the TF-AKT/ERK pathway.

## DISCUSSION

TBI stands as a primary cause of long-term disabilities among adolescents [31]. Worldwide, around 70 million individuals experience TBI annually, with treatment costs exceeding $400 billion [29, 32], posing a substantial challenge to socio-economic realms and posing a weighty public health concern [7]. From the perspectives of pathology and etiology, one of the principal complications accompanying therapeutic approaches to traumatic brain injuries is the intricacy of their underlying mechanisms. Presently, it is recognized that primary injuries resulting from mechanical forces are seldom curable; however, there exists potential for ameliorating and reversing secondary injuries subsequent to TBI. This has spurred an escalating focus on the issue in recent years [33]. In this study, we introduced a breakthrough discovery: tDCS promotes angiogenesis at the injury site by upregulating the OXA-TF-AKT/ERK signaling pathway, thereby contributing to neurological recovery after brain trauma. Additionally, we unearth that tDCS holds the capacity to counteract blood-brain barrier disruption subsequent to traumatic brain injuries, an effect achieved by upregulating tight junction and adhesion proteins.

tDCS has been established as an effective therapeutic approach for TBI, with research indicating its absence of side effects [34, 35]. In terms of its therapeutic mechanism, specifically, anodal current exerts an excitatory effect on neuronal activity, while cathodal tDCS exhibits inhibitory properties [36]. A study has highlighted the ability of anodal tDCS to enhance reaction times in working memory tasks for patients with mild to moderate brain injuries, providing crucial therapeutic insights for their recovery [37]. Furthermore, anodal tDCS has demonstrated the potential to promote cerebrovascular reactivity in cerebral parenchymal arterioles, regulating average cerebral blood flow and tissue oxygenation in traumatized mice, thus potentially contributing to the improvement of neurological function [15, 38]. Despite being considered an effective therapeutic modality for TBI, the molecular mechanisms governing the effects of tDCS are still not fully understood [36]. Hence, in this research, we proceeded to examine the impact of tDCS on vascular regeneration and angiogenesis in a rat model of TBI. Our research findings demonstrate a significant reduction in vascular density near the site of TBI, accompanied by a corresponding activation of angiogenesis (CD31+Brdu double-positive cells), consistent with the results of Wu et al. [7, 39]. However, following 2 weeks of tDCS treatment, there is a remarkable increase in vascular density at the injury site, along with a substantial increase in the proportion of newly formed blood vessels. Concurrently, there is a significant improvement in neurobehavioral function in TBI rats. Notably, this effect can be attenuated by the Orexin-A receptor antagonist SB334867. These results further validate the effectiveness of tDCS in treating TBI and its association with angiogenesis and Orexin-A.

The process of vascular neogenesis is controlled by the expression of a variety of angiogenic growth factors and regulators, with the most prevalent and crucial regulator being VEGF [7, 40]. In animal models of TBI, VEGF expression is upregulated within 3-7 days post-injury [40]. The VEGF family encompasses numerous members, and among them, VEGFA plays a vital role in coordinating the process of angiogenesis [41]. We assessed the levels of VEGFA protein expression at the site of injury and found that on the 14th day post-TBI, there was a decrease in VEGFA levels, contrary to some previous research findings. We hypothesize that this variation could be due to differences in the severity of the injury, the site of sample collection, and the timing of evaluation. Notably, following tDCS treatment, VEGFA expression was upregulated, along with a concurrent increase in CD31 expression, and this therapeutic effect could be suppressed by SB334867. Additionally, we evaluated the mRNA expression levels of angiogenic factors VEGFA and CD31 in brain tissues at the injury site. The results revealed reduced expression levels of VEGFA and CD31 in the TBI group, which saw significant restoration after tDCS treatment. Similarly, this effect could also be inhibited by SB334867. This suggests that tDCS can upregulate the expression of VEGFA to promote angiogenesis and is closely associated with Orexin-A.

Bleeding, swelling, irregular blood flow, and the breakdown of the BBB are typical immediate and early occurrences seen in both patients with TBI and animal models of TBI [42]. The correlation between sustained BBB dysfunction following TBI has been well-established. BBB disruption occurs within hours after brain injury and may persist for years, often associated with a poor prognosis [43]. Physical disruption of the BBB due to injury results in the penetration of plasma electrolytes and proteins (vasogenic edema), which then enter intracellular spaces (cytotoxic edema). Consequently, early edema develops within 1–12 hours after the injury [44]. Early control of brain edema contributes to the secondary brain tissue repair process, mitigates secondary brain injury, and reduces neurological deterioration and mortality rates [45]. TJ and AJ proteins play a critical role in maintaining BBB integrity [7]. In this study, we observed significant BBB impairment following TBI, characterized by structural damage, pronounced brain edema, and downregulation of brain microvascular tight junction proteins (ZO-1, occludin, VE-Cadherin). After 2 weeks of tDCS treatment, BBB damage induced by TBI was significantly attenuated, accompanied by reduced Evans blue extravasation and substantial relief of brain edema, ultimately leading to improved neurological function in rats. Importantly, this protective effect could be diminished by SB334867. These results indicate that tDCS can ameliorate BBB disruption and brain edema resulting from TBI, and this therapeutic effect is closely associated with Orexin-A.

Orexin-A is a neuropeptide that regulates various physiological functions, including sleep/wake cycles, appetite, energy metabolism, and neuroendocrine processes, through its receptors OX1R (Orexin type 1 receptors) and OX2R (Orexin type 2 receptors) [46]. Our earlier studies have demonstrated that Orexin-A can alleviate brain tissue damage and significantly improve neurobehavioral function in rats following TBI [47], consistent with the findings of Adamantidis et al. [48]. Furthermore, Orexin-A has been identified as a novel angiogenic peptide, promoting blood vessel formation by activating the MEK/ERK1/2 signaling pathway through Orexin-A receptor activation [12], aligning with our research results. In this study, we conducted *in vitro* and *in vivo* Matrigel plug experiments to investigate the effects of Orexin-A on HUVEC cell migration and tube formation. These results unequivocally demonstrate the promotive effect of Orexin-A on endothelial cell migration and vascular formation. Importantly, we further supported these findings by using an ERK1/2 inhibitor, elucidating the intricate interplay between Orexin-A-driven angiogenesis and cell migration via the OX1R/ERK1/2 pathway. Moreover, the TF/AKT/ERK pathway has been firmly established as closely related to angiogenesis, further emphasizing the mechanistic significance of Orexin-A in the process of vascular neogenesis. [29, 30]. Further investigations involving various endothelial cell lines are planned for future studies. In our animal experiments, a significant downregulation of OX1R-TF-AKT/ERK pathway-related proteins was observed at the injury site in the TBI group. However, subsequent tDCS treatment resulted in a notable upregulation of these proteins. Importantly, this therapeutic effect could be significantly inhibited by the Orexin-A receptor antagonist SB334867. Furthermore, the expression levels of these pathway proteins correlated with changes in injury site vascular density and rat neurobehavioral function. These findings suggest that tDCS promotes vascular regeneration at the injury site through modulation of the OX1R-TF-AKT/ERK pathway, thereby contributing to therapeutic effects on neurobehavioral recovery.

## CONCLUSIONS

In summary, our study has demonstrated several key findings: (a) tDCS can promote brain tissue repair and improve neurological function in rats following TBI; (b) tDCS achieves this by activating the OXA-TF-AKT/ERK signaling pathway, facilitating post-TBI vascular neogenesis and aiding in the recovery of lost blood vessels. Furthermore, *in vitro* experiments have further confirmed the promoting effect of Orexin-A on vascular formation, closely associated with the OXA/ERK/1/2 signaling pathway; (c) tDCS can upregulate the expression of TJ and AJ proteins, contributing to the rescue of the BBB disruption and alleviating brain edema following TBI. Therefore, we believe that tDCS holds potential in the treatment of TBI by promoting vascular neogenesis and enhancing neurological function. However, it is worth noting that our present research, certain constraints or shortcomings persist. The factors influencing post-TBI vascular neogenesis are highly complex, and our study only partially elucidated the mechanism through which tDCS promotes vascular generation by activating the OXA-TF-AKT/ERK signaling pathway. Additionally, the interactions between tDCS in regulating vascular repair and neural recovery remain unclear and warrant further in-depth investigation.
